# Inhibition or Stimulation of Ochratoxin A Synthesis on Inoculated Barley Triggered by Diffuse Coplanar Surface Barrier Discharge Plasma

**DOI:** 10.3389/fmicb.2018.02782

**Published:** 2018-11-16

**Authors:** Julia Durek, Oliver Schlüter, Anne Roscher, Pawel Durek, Antje Fröhling

**Affiliations:** ^1^Leibniz Institute for Agricultural Engineering and Bioeconomy, Quality and Safety of Food and Feed, Potsdam, Germany; ^2^German Rheumatism Research Centre Berlin, Berlin, Germany

**Keywords:** mycotoxin, ochratoxin A, *Aspergillus niger*, Penicillium verrucosum, cold atmospheric plasma, mold inhibition

## Abstract

Ochratoxin A (OTA) is one of the most abundant food-contaminating mycotoxins. Besides their high toxicity, mycotoxins are highly stable to physical, chemical or biological detoxification. Therefore, the treatment with cold atmospheric plasma could be one approach to reduce the amount of mycotoxins in different products. The aim of this study was to determine the influence of cold atmospheric plasma on the inactivation of *Aspergillus niger* and *Penicillium verrucosum* inoculated on barley and their production of OTA. Inoculated barley was treated with plasma generated by dry air, CO_2_ or CO_2_ + O_2_ for 1 or 3 min and stored for up to two weeks at 9, 25, or 37°C. Three minutes of air plasma treatment effectively significantly reduced the total mold count of both microorganisms by 2.5–3 log cycles. The production of OTA from *A. niger* was only low, therefore the treatment effect was indistinguishable. The treatment of *P. verrucosum* on barley after an incubation of five days using a CO_2_ + O_2_ plasma resulted in a reduction of the OTA content from 49.0 (untreated) to 27.5 (1 min) and 23.8 ng/g (3 min), respectively. In contrast, CO_2_ plasma caused an increase of the OTA amount from 49.0 (untreated) to 55.8 (1 min) and 72.9 ng/g (3 min). Finally, the use of air plasma resulted likewise in a decrease of the OTA concentration from 56.9 (untreated) to 25.7 (1 min) and 20.2 ng/g (3 min), respectively. Reducing the incubation time before the treatment to 24 h caused in contrast an increase of the OTA content from 3.1 (untreated) to 29.1 (1 min) and 20.7 ng/g (3 min). Due to the high standard deviation, these changes were not significant, but the tendencies were clearly visible, showing the strong impact of the plasma gas on the OTA production. The results show, that even if the total mold count was reduced, under certain conditions the OTA amount was yet enhanced, probably due to a stress reaction of the mold. Concluding, the plasma gas and incubation conditions have to be considered to allow a successful inactivation of molds and in particular their toxic metabolites.

## Introduction

When crop is not stored properly after harvest, especially when moisture content and temperature are too high, different molds like, e.g., *Aspergillus niger* and *Penicillium verrucosum* can grow on its surface. Both molds belong to the family Trichocomaceae. Family members are saprobes with aggressive colonization strategies, adaptable to extreme environmental conditions. They are cosmopolitan in distribution, ubiquitous in soil, and common associated with decaying plant and food material. *Aspergillus niger* (*A. niger*) is one of the most common species of the genus *Aspergillus* and is a frequent contaminant of food. The mold grows at temperatures between 6 and 47°C, with a temperature optimum between 35 and 37°C. The spores do not survive damp cold at −22°C or less. The mold tolerates pH ranges from 1.5 to 9.8 and is therefore able to exist in both strongly acidic and basic environments. Some strains of *A. niger* can produce the mycotoxin ochratoxin A (Abarca et al., 1994). *Penicillium verrucosum* (*P. verrucosum*) belongs to the genus *Penicillium* and has important implications in food, specifically for grains and other cereal crops on which it grows predominantly in Northern Europe. It has a white mycelium with green conidia, which have the ability to germinate at temperatures between 0 and 31°C with a temperature optimum between 21 and 23°C (Domsch et al., 1980). At temperatures between 10 and 25°C and a water activity (a_w_) of around 0.95, ochratoxin A (OTA) synthesis of some *P. verrucosum* strains occur (Lund and Frisvad, 2003; Cairns-Fuller et al., 2005).

Due to its chemical stability against heat and during industrial food processing, OTA is one of the most abundant food-contaminating mycotoxins. It is a naturally occurring mycotoxin and a secondary metabolite of toxigenic species of *Aspergillus* and *Penicillium* molds, e.g., *A. niger* or *P. verrucosum*. It is present in different geographical regions and contaminates cereals such as wheat, maize, rye, barley, and oats under preharvest and postharvest conditions. It also occurs in peanuts, coffee beans, bread, rice, and dried fruits (Kuiper-Goodman and Scott, 1989; Al-Anati and Petzinger, 2006). When ingested in the organism, OTA can be found in various tissues, with particularly high accumulation in the kidney. As a result, the compound has predominantly nephrotoxic, but also hepatotoxic, immunotoxic and possibly neurotoxic properties. It has also been classified as probably carcinogenic for humans (Kuiper-Goodman and Scott, 1989; IARC Monographs, 1993). The exact mechanism that leads to toxicity is not yet fully understood. Due to its high toxicity, the maximum level of OTA in different foods is regulated, e.g., in unprocessed cereals to 5 μg/kg (FAO, 2006).

In general, mycotoxins including OTA are highly stable to physical, chemical or biological detoxification. For example, the thermal stability of OTA depends on the water content. When heating dry milled wheat, the reduction in OTA content only occurred at high temperatures and long exposure times (e.g., 50% reduction at 150°C and 200 min), while the reduction was slightly faster with moistened material; however, complete destruction of OTA was not achieved even at 200 or 250°C, respectively (Boudra et al., 1995). Even a gamma irradiation (20 kGy) of feed contaminated with OTA resulted only in a reduction of 36–47% (Refai et al., 1996). A treatment of barley with 5% ammonia for 96 h at 70°C resulted in a 90% reduction, but feeding the barley showed only a slight improvement in pig performance over contaminated cereals (Madsen et al., 1983). Mixing of contaminated barley with 3.5% NaOH for 30 min reduced the OTA amount from approximately 650 to 20–60 μg/kg (Richter et al., 1997), but the OTA reaction with NaOH was reversible (Valenta and Richter, 1998).

A relatively new method for a product-protecting decontamination of contaminated food could be a cold plasma treatment at atmospheric pressure, which is mostly of interest for the food industries due to the used moderate conditions (Misra et al., 2011). The types of plasma generation contain the corona discharge, radio-frequency plasmas, the gliding arc discharge and dielectric barrier discharges. The inactivation of microorganisms is based on different mechanisms. Resulting reactive oxygen- and nitrogen-based species have a direct oxidative effect on the outer cell surface (‘etching’), provoking lesions which are not reparable fast enough. Additionally, UV irradiation can directly damage the genetic material of the microorganisms. Furthermore, intrinsic photodesorption by UV photons may occur, destroying chemical cell bonds and releasing volatile compounds of intrinsic atoms of the microorganism (Moisan et al., 2001).

For several years, plasma applications were used for sterilization of food and other products (Moisan et al., 2001; Laroussi, 2005; Niemira, 2012). Plasma was also found to be effective in reducing molds on food surfaces (Hojnik et al., 2017). The treatment of, e.g., maize grains contaminated with *A. flavus* und *A. parasiticus* spores resulted in a decrease of 5.5 and 5.2 log CFU/g after 5 min in a non-thermal atmospheric pressure fluidized bed plasma system with air as plasma gas (Dasan et al., 2016). Moreover, plasma has also the potential to inactivate different mycotoxins, like, e.g., aflatoxin B1, deoxynivalenol (DON) and nivalenol, that were degraded after 5 s of treatment with a microwave-induced argon plasma system at atmospheric pressure (Park et al., 2007). Using ambient air as plasma gas, deoxynivalenol, zearalenone, enniatins, fumonisin B1, T2 toxin, and sterigmatocystin were completely degraded within 60 s. Zearalenone, enniatin B, fumonisin B1, and sterigmatocystin were additionally embedded in mold cultures on rice to investigate the matrix effects. For zearalenone and sterigmatocystin, the degradation rates were slowed down, but after 60 s, nearly the full amount was removed. For enniatin B and fumonisin B1 instead, nearly half of the mycotoxins remained intact after 60 s (ten Bosch et al., 2017). On malting barley, DON was reduced to ca. 82% of the initial value and trichothecene (T-2) decreased to 40% of the initial content after 4 min treatment with a gliding arc discharge at atmospheric pressure (Kříž et al., 2015).

However, only one study considered the effect of plasma on the actual production of mycotoxins by molds. Ouf et al. (2015) contaminated date palm fruits with *A. niger* and treated them i. a. for 7.5 min with a double atmospheric pressure cold argon plasma, which resulted in a complete reduction of OTA after 10 days at 25°C. However, as argon is a noble gas and therefore relatively expensive and the treatment time with 7.5 min quite long, in this study different cheaper plasma gasses were tried to have a similar effect. Therefore, in this study, barley was inoculated with *A. niger* and *P. verrucosum*, then treated with cold plasma generated by dry air, CO_2_ and 80% CO_2_ + 20% O_2_ and stored for up to two weeks to observe the influence on the inactivation of molds and their production of OTA on barley.

## Materials and Methods

### Inoculation of Barley

The experiments were performed with barley of the variety ‘Grace 1250’ (IREKS, Kulmbach, Germany). The barley had an initial moisture content of 10.5–11% and was sterilized in an autoclave at 134°C for 20 min.

As molds *Aspergillus niger* (DSM 22593) and *Penicillium verrucosum* (provided by Max Rubner-Institut, Karlsruhe, Germany) were used. They were spread on Potato-Dextrose-Agar (PDA) and grown for five days at 37°C (*A. niger*) or seven days at 25°C (*P. verrucosum*), respectively.

For the inoculation of the barley, five agar pieces including mycelium (cut out with a cork borer Ø = 1 cm) were mixed with 3 mL purified water to get a mycelium suspension. 1.9 mL of this suspension was used to inoculate 10 g of autoclaved barley in a 50 mL tube resulting in a final moisture content of 25–28%, corresponding to a_w_ values of 0.949–0.968 in the barley, which was adequate for the used molds to induce the production of mycotoxins (Fleurat-Lessard, 2017). Over the storage period, the barley was not moistened again and therefore, moisture content probably decreased. After homogeneous mixing for 1 h in an overhead shaker, the barley was spread evenly distributed into a petri dish and incubated at 37°C (*A. niger*) or 25°C (*P. verrucosum*) overnight in an incubator. On the next day, the plasma treatment was performed.

In addition, for one series of experiments with *A. niger* using plasma generated with air, a spore suspension was used for inoculation of the barley. For the preparation of the spore suspension, 10 mL of ringer solution (Merck KGaA, Darmstadt, Germany) was spread on the PDA plate, overgrown with *A. niger*. Subsequently, the surface was rubbed by an inoculation loop to detach the mycelium including spores. The resulting suspension was filtered afterward through a gauze bandage to remove pieces of mycelium. The concentration of the spore suspension was counted using the THOMA chamber and varied between 5.59 and 5.78 log spores/g barley.

Furthermore, for another series of experiments with *P. verrucosum* using plasma generated with air, CO_2_ or 80% CO_2_ + 20% O_2_, the incubation time of the inoculated barley before the plasma treatment was extended to five days to allow an adaption of the mold to the barley.

For a better overview, the performance of the experiments is described in Table 1.

**Table 1:** Performance of the realized experiments with all varied parameters.

Strain	Inoculation method	Incubation time before plasma treatment	Incubation temperature	Plasma treatment	Storage time after plasma treatment	Storage temperature	Measured parameters
*A. niger*	Mycelium suspension	24 h	37°C	Dry air; CO_2_; CO_2_+O_2_	1 week	37°C	OTA
*A. niger*	Mycelium + spore suspension	24 h	37°C	Dry air	2 weeks	9°C	OTA; CFU/g
*P. verrucosum*	Mycelium suspension	110 h	25°C	Dry air; CO_2_; CO_2_+O_2_	2 weeks	25°C	OTA
*P. verrucosum*	Mycelium suspension	24 h	25°C	Dry air	2 weeks	9°C	OTA; CFU/g

### Plasma Source and Treatment

A diffuse coplanar surface barrier discharge (DCSBD) 400 plasma source (CEPLANT, R&D Centre for Low-Cost Plasma and Nanotechnology Surface Modifications, Masaryk University, Brno, Czechia) was used for the plasma treatment of the barley. The plasma equipment is described in detail by Hertwig et al. (2017). The treatment was performed in a reaction chamber with one bottom and one top DCSBD plate, the last one being adjustable in height. Between the plates, a plastic mesh with a distance of 1.5 cm to the plates was placed, where 20 g of barley were evenly spread. Plasma was only generated in the top plate; the power input was set to 350 W. For the plasma generation, dry air, CO_2_ or 80% CO_2_ + 20% O_2_, regulated by a gas flow controller (Multi Gas Controller 647C, MKS Instruments, Andover, MA, United States), were used as process gasses with a gas flow of 10 sL/min. After flushing the reaction chamber with the respective process gas, plasma treatment for 1 or 3 min occurred, followed by another flushing step to remove the generated residues. The experiments were performed in triplicates. After plasma treatment, the inoculated barley was stored at 9°C (*P. verrucosum* and *A. niger*), 25°C (*P. verrucosum*) or 37°C (*A. niger*) for up to two weeks.

### Microbiological Analysis

For microbiological analysis of mold count on the inoculated barley, 5 g of barley were transferred into a sterile flask. A 1:10 dilution was prepared using peptone salt solution (DIN EN ISO 6887-1:1999) and the mixture was shaken for 30 min at 180 rpm (TR-125, Infors AG, Bottmingen, Switzerland) to allow a removal of molds from the barley. Subsequently, the samples were serially diluted in Rotilabo^®^-microtest plates (96er U-profile, Carl Roth GmbH & Co. KG, Karlsruhe, Germany) using peptone salt solution. 100 μl of each dilution were spread on Potato-Dextrose-Agar (AppliChem GmbH, Darmstadt, Germany) in duplicates. After growth at 25°C (*P. verrucosum*) or 37°C (*A. niger*) for up to 72 h, the number of colony forming units (CFU/g) was determined to obtain the aerobic viable mold count of the barley. The lower detection limit of the plate count analyses was 100 CFU/g. The total mold counts for different conditions were compared using Welch’s unequal variances *t*-test. *P*-values below 0.05 were considered statistically significant.

### HPLC Analysis

For the extraction of ochratoxin A, ca. 2 g of ground (45 s, Superior PCML-2013A, Harvest Industry Limited, Guangzhou, China) barley was mixed with 10 mL acetonitrile (80%, VWR International GmbH, Dresden, Germany) with glacial acetic acid (1%, Carl Roth GmbH & Co. KG, Karlsruhe, Germany) for 60 min in an overhead shaker (Stuart^®^ Rotator STR4, Bibby Scientific Ltd., Staffordshire, United Kingdom). Afterward, the samples were centrifuged (Centrifuge 5810 R, Eppendorf AG, Hamburg, Germany) at 3100 × *g* for 10 min and the supernatant was filtered (1 μm). A mixture of 5 mL filtrate and 35 mL aqua dest was cleaned up on an immunoaffinity column (OchraTest WB, Ruttmann GmbH, Hamburg, Germany). After rinsing with 5 mL aqua dest, OTA was eluted with 1.5 mL acetonitrile (100%) + glacial acetic acid (1%) and evaporated at 60°C in an evaporator (Concentrator Plus, Eppendorf AG, Hamburg, Germany) to dryness. The residue was redissolved in 0.2 mL acetonitrile (70%) + glacial acetic acid (1%), which was also used for preparation of the OTA-standard for calibration of the HPLC. The used HPLC unit (Nexera^TM^ HPLC, Shimadzu Deutschland GmbH, Duisburg, Germany) was equipped with a pump (LC-30 AD), an auto sampler (SIL-30 AC), a degasser (DGU- 20A5), a column oven (CTO 20 AC), a fraction collector (FRC-10A), a fluorescence detector (FLD, RF-20A XS) and LabSolution software. The column was a reverse-phase column (EC 250/4.6 Nucleodur Sphinx RP, 3 μm, Macherey-Nagel, Düren, Germany) with a reverse-phase pre-column (XBridge BEH Shield RP 18, 3.5 μm, Waters GmbH, Eschborn, Germany) and the fluorescence detector was adjusted to λ_ex_/λ_em_ = 333/460 nm. The calibration curve for OTA ranged from 1 to 100 ng/mL (*r*^2^ = 0.999) and the injection volume of the samples was 10 μL. The limit of detection was 0.5 ng/mL and the limit of quantification was 1 ng/mL. The method was adapted according to DIN EN (14132):2009 (2009). The OTA amounts for different conditions were compared using Welch’s unequal variances *t*-test. *P*-values below 0.05 were considered statistically significant.

## Results and Discussion

### Plasma Treatment of Barley, Inoculated by *Aspergillus niger*

**FIGURE 1:**
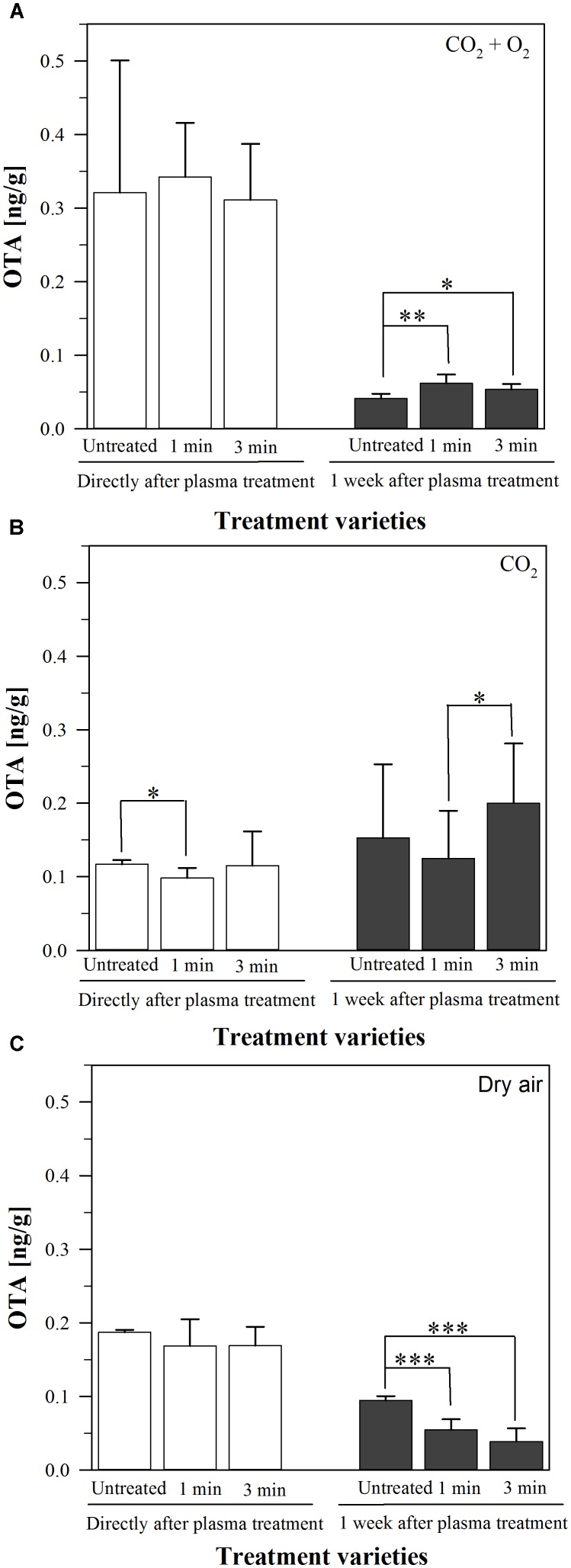
Amount of ochratoxin A (OTA, [ng/g]) **(A–C)** on barley, inoculated by a mycelium suspension of *A. niger*, directly or one week after plasma treatment for 1 or 3 min or without any treatment, stored at 37°C. Significance levels ^∗^*p* < 0.05; ^∗∗^*p* < 0.01; ^∗∗∗^*p* < 0.001.

After inoculation of barley with a mycelium suspension of *Aspergillus niger* followed by a plasma treatment with air, CO_2_ or 80% CO_2_ + 20% O_2_ as process gasses for 1 or 3 min, the amount of produced OTA directly and after storage at 37°C for one week was measured using HPLC. This storage temperature was chosen, because it is the optimal growth temperature of *A. niger*. The measured amounts were all under 0.36 ng/g, one week after the treatment they had decreased to <0.25 ng/g (Figure 1). One exception was the plasma treatment using CO_2_ as process gas for 3 min, where the OTA concentration slightly increased after one week from 0.15 ± 0.1 ng/g in the untreated grains to 0.20 ± 0.08 ng/g after 3 min plasma. However, this increase was not significant; and in general, these low amounts cannot be attributed alone to the plasma treatment since the untreated barley showed similar values.

**FIGURE 2:**
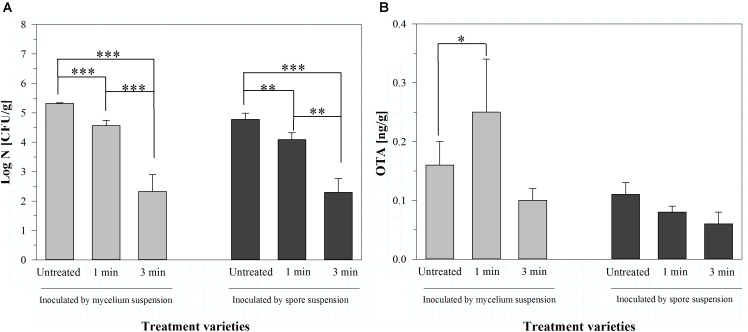
Total mold count [log CFU/g] **(A)** and amount of ochratoxin A (OTA, [ng/g]) **(B)** on barley, inoculated with *A. niger* as mycelium or spore suspension, two weeks after air plasma treatment for 1 or 3 min or without any treatment, stored at 9°C. Significance levels ^∗^*p* < 0.05; ^∗∗^*p* < 0.01; ^∗∗∗^*p* < 0.001.

Even if the concentration of OTA in general was low, significant inactivation rates were achieved using dry air as process gas. Therefore, the experiments were repeated using only dry air as process gas, but with two different inoculation methods (mycelium and spore suspension) and a lower storage temperature of 9°C. This storage temperature was chosen to investigate the effect of a lower temperature on the growth of the molds and the production of the mycotoxins, because the latter can be higher at lower temperatures (Sherwood and Peberdy, 1974). Furthermore, the success of the inoculation and the effect of the plasma treatment on the mold were controlled by analyzing the total mold count of the barley two weeks after the plasma treatment, because there was no mycelium visible at this storage temperature (Figure 2).

The inoculation with a mycelium suspension resulted in a total mold count of 5.32 ± 0.02 log CFU/g. The treatment with air plasma for 1 min significantly reduced the total mold count to 4.57 ± 0.18 log CFU/g and the treatment for 3 min lowered it to 2.32 ± 0.58 log CFU/g. The corresponding amounts of OTA were 0.16 ± 0.04 ng/g for the untreated barley, 0.25 ± 0.09 ng/g after 1 min plasma and 0.10 ± 0.02 ng/g after 3 min plasma treatment, showing no significant difference. Summarizing, the treatment with plasma for 1 min resulted in a slight, but not significant increase of the OTA amount, compared to the untreated barley. Prolonging the time of plasma treatment led in contrast to a slight, but also not significant decrease of OTA in the barley (Figure 2).

The inoculation of the barley with a spore suspension resulted in slightly lower total mold counts compared to the inoculation by mycelium suspension (Figure 2A). However, the air plasma treatment caused a similar, significant reduction [from 4.78 ± 0.21 log CFU/g (untreated) to 4.09 ± 0.23 log CFU/g (1 min plasma) and 2.29 ± 0.47 log CFU/g (3 min plasma)] of the total mold count. According to the lower total mold count, also the amount of OTA in the barley was slightly lower (0.11 ± 0.02 ng/g). Treatment with plasma for 1 min caused a slight decrease to 0.08 ± 0.01 ng/g, and 3 min of plasma treatment nearly halved the OTA amount in the barley (Figure 2B). However, the OTA amounts in the barley produced by *A. niger* showed no significant differences after the treatments. In general, the OTA contents were again low, as in consequence the validity of the plasma treatment regarding the influence on the OTA production at inoculated barley.

Comprising, the reduction of the total mold count after air plasma treatment for 1 min was 0.75 log CFU/g (mycelium suspension) or 0.69 log CFU/g (spore suspension), respectively. The plasma treatment for 3 min caused a decrease of 3 log CFU/g (mycelium suspension) or 2.49 log CFU/g (spore suspension), respectively. It is assumed that the total mold counts were even lower directly after plasma treatment, because during 2 weeks of storage the microorganisms would obviously have more time to grow. This reduction of the total mold counts was in good accordance with other results. Using a low pressure cold plasma prototype unit with air as process gas, *Aspergillus* spp. on seeds was reduced by around one log cycle after 5 min (Selcuk et al., 2008). *Aspergillus parasiticus* on hazelnuts was reduced by around 2.5 log cycles after 3 min treatment by an atmospheric pressure fluidized bed plasma using dry air as process gas (Dasan et al., 2017). Many reactive oxygen and nitrogen species like, e.g., nitrous gasses and also UV photons are generated in air plasmas (Laroussi and Leipold, 2004; Laroussi, 2005; Hertwig et al., 2017). The resulting oxidative effects lead to strong damages on fatty acids and proteins in the cell membranes and on the genetic material (Laroussi and Leipold, 2004; Boudam et al., 2006). Additionally, erosion of the microorganisms through intrinsic photodesorption and etching occurs (Moisan et al., 2001).

### Plasma Treatment of Barley, Inoculated by *Penicillium verrucosum*

*Penicillium verrucosum* was used for inoculation of the barley to see the effect of the plasma treatment on another mold also producing OTA as mycotoxin. Barley was inoculated with *P. verrucosum*, incubated for 5 days, treated by plasma generated with different process gasses for 1 or 3 min and stored at 25°C for two weeks. This temperature was the optimal growth temperature for the mold. The incubation time of *P. verrucosum* on the barley was longer in order to achieve a certain amount of mycotoxins on the samples to allow the investigation of mycotoxin inhibition by cold plasma treatment.

**FIGURE 3:**
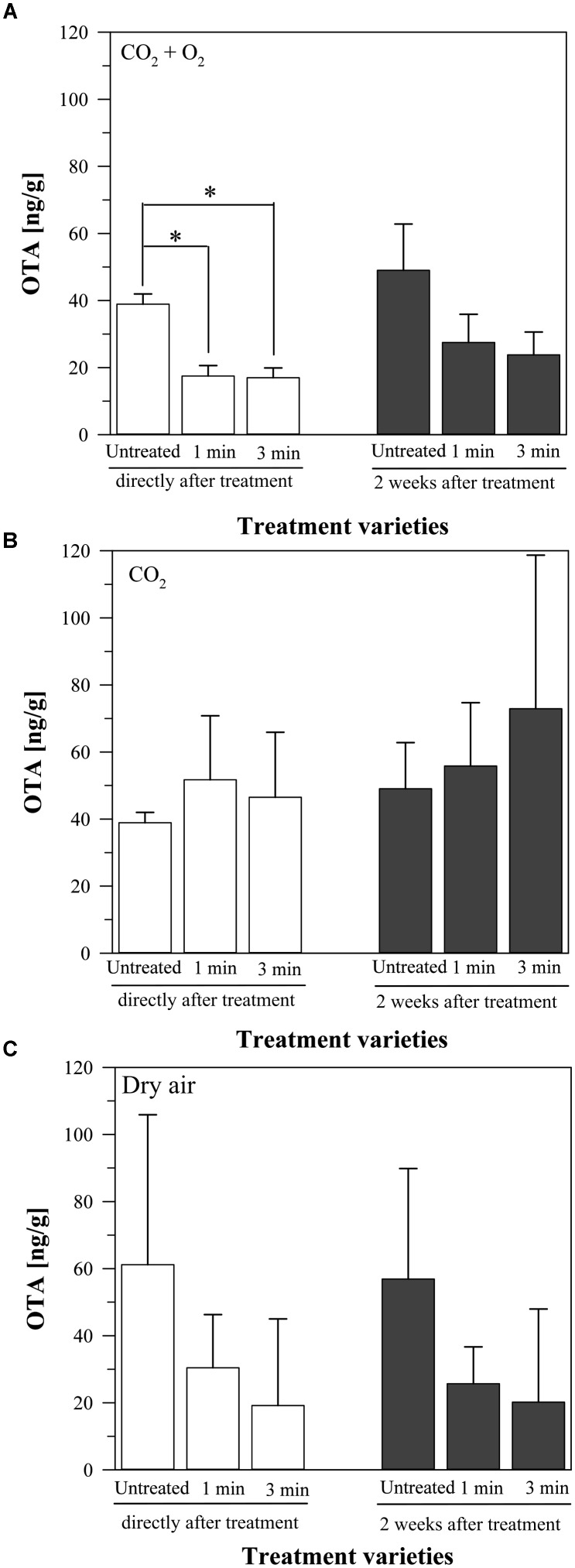
Amount of ochratoxin A (OTA) on barley, inoculated with a mycelium suspension of *P. verrucosum*, directly or two weeks after plasma treatment for 1 or 3 min or without any treatment, stored at 25°C. Process gasses: CO_2_ + O_2_ **(A)**, CO_2_ **(B)**, and dry air **(C)**. Significance level ^∗^*p* < 0.05.

When using a mixture of CO_2_ + O_2_ as plasma process gas, the production of OTA on the inoculated barley was reduced compared to the untreated grains (38.9 ± 3.1 ng/g, Figure 3A). Plasma treatment for 1 min significantly reduced OTA to 17.5 ± 3.1 ng/g and for 3 min to 17 ± 2.9 ng/g. Two weeks after treatment the amount of OTA slightly increased in all cases. The untreated grains showed now an OTA content of 49 ± 13.8 ng/g. After 1 min plasma treatment, the OTA amount increased to 27.5 ± 8.4 ng/g and after 3 min plasma to 23.8 ± 6.8 ng/g. Compared to the untreated barley, the OTA content was therefore still reduced to around the half, albeit this decrease was not significant due to the high standard deviation.

In contrast, the use of only CO_2_ as plasma process gas had a different effect on the OTA production (Figure 3B). Compared to the untreated barley (38.9 ± 3.1 ng/g OTA), the plasma treatment for 1 min resulted in an increase (51.7 ± 19.1 ng/g OTA), which was a little lower after 3 min plasma treatment (46.5 ± 19.4 ng/g OTA). After 2 weeks of storage, the OTA content increased again in all varieties. The OTA amount of the untreated grains was now 49 ± 13.8 ng/g, the OTA content of the 1 min plasma treated grains was 55.8 ± 18.9 ng/g, and the one of the 3 min plasma treated barley was 72.9 ± 45.8 ng/g. Compared to the untreated barley, the treatment varieties caused all an increase of the OTA production of *P. verrucosum*. However, this increase was not statistically significant due to the high standard deviation of the results.

The treatment with air plasma caused again a decrease of the OTA amounts. At the untreated barley, the OTA content was 61.2 ± 44.7 ng/g, compared to 30.4 ± 15.9 ng/g after 1 min plasma and 19.2 ± 25.8 ng/g after 3 min plasma, analyzed directly after treatment (Figure 3C). Two weeks later, the values were nearly the same. The OTA amounts in the untreated grains slightly decreased to 56.9 ± 33 ng/g, like in the 1 min plasma treated barley (to 25.7 ± 11 ng/g OTA). The values for the 3 min treatment slightly increased to 20.2 ± 27.7 ng/g OTA. However, the standard deviation of the results was again too high to reveal a significant difference.

In dielectric barrier discharges generated with air or oxygen, especially ozone and reactive oxygen species are formed (Eliasson et al., 1987; Kalghatgi et al., 2012; Kogelschatz, 2012).

In particular, the formation of ozone in plasmas generated with O_2_ as process gas is 10-fold higher than in CO_2_ plasmas (Hertwig et al., 2017). Ozone has a high antimicrobial potential due to the occurring oxidation of cell components like polyunsaturated fatty acids, enzymes and proteins (Victorin, 1992). The high amount of ozone in the plasmas generated by CO_2_ + O_2_ and dry air led therefore probably to a stronger inactivation of *P. verrucosum*, resulting in a reduced ability to produce OTA. Additionally, ozone has a direct inactivating effect on the pre-existing mycotoxins by causing chemical modifications leading to a reduced biological activity (Tiwari et al., 2010). Due to the long incubation time before the plasma treatment, the mold produced a high amount of OTA, which was then attackable by the ozone. In the case of using only CO_2_ as process gas, the formation of ozone was lower, probably leading to a minor damage of the mold and the pre-existing OTA.

In another experiment, the incubation time before the plasma treatment was shortened to investigate the influence of plasma treatment directly on the production process of mycotoxins. Additionally, the total mold count of the inoculated barley was recorded in order to determine the direct effect of the plasma treatment on *P. verrucosum*. Therefore, barley was inoculated with *P. verrucosum*, incubated for 24 h, treated with plasma generated by dry air for 1 or 3 min and stored for one or two weeks at 9°C. The lower temperature could be an additional stress factor for the mold that may affect the mycotoxin production.

**FIGURE 4:**
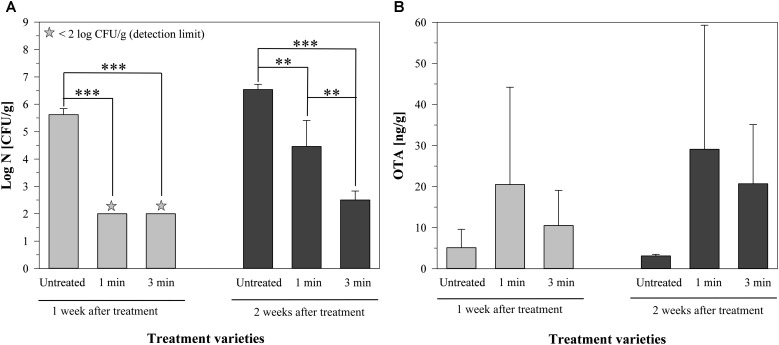
Total mold content **(A)** and amount of ochratoxin A **(B)** on barley, inoculated with a mycelium suspension of *P. verrucosum*, 1 and 2 weeks after air plasma treatment for 1 or 3 min or without any treatment, stored at 9°C. Significance levels ^∗∗^*p* < 0.01; ^∗∗∗^*p* < 0.001.

The total mold count of the untreated barley was 5.62 ± 0.22 log CFU/g after one week, increasing to 6.54 ± 0.19 log CFU/g after two weeks of storage (Figure 4A). The air plasma treatment for 1 min led to a decrease of the total mold count under the detection limit of 2 log CFU/g after one week of storage, followed by another increase to 4.46 ± 0.95 log CFU/g after two weeks. A longer plasma treatment for 3 min resulted in a reduction below the detection limit after one week and a total mold count of 2.50 ± 0.33 log CFU/g after two weeks of storage (Figure 4A).

Looking at the respective amounts of OTA, the untreated grains had a concentration of 5.1 ± 4.5 ng/g after one week of storage, which was slightly lower (3.1 ± 0.4 ng/g) after two weeks (Figure 4B). One minute of air plasma resulted in an OTA content of 20.5 ± 23.7 ng/g or 29.1 ± 30.2 ng/g after one or two weeks, respectively. The plasma treatment for 3 min led to an increase to 10.5 ± 8.6 ng/g after one week and to 20.7 ± 14.4 ng/g after two weeks of storage. The standard deviation was relatively high and therefore the differences were not significant, but it was still visible, that the amount of OTA increased after the treatment with air plasma, even if *P. verrucosum* was inactivated to some extent by the treatments (Figure 4), which implies that the plasma treatment with air has led to a triggering of the mycotoxin production.

In other studies, the spores of *Penicillium expansum* were reduced by corona discharge plasma for approx. one log CFU/mL after 120 min (Ye et al., 2012). Electron micrographs revealed noticeable defects in the morphology and internal sub-structure of the spores, leading to the inactivation. Spores in general are highly resistant against all kinds of disinfection, which also explains the long inactivation time. On dried filefish fillets, *P. citrinum* was reduced by 1 log CFU/g after 10 min of cold oxygen plasma treatment (Park and Ha, 2014). *Penicillium* spp. on grain was likewise decreased for 1 log cycle after 5 min of air plasma treatment (Selcuk et al., 2008).

Other research showed that the use of neutralized electrolyzed water reduced the Fusarium microbial count on wheat, but partially increased the amount of produced deoxynivalenol (Audenaert et al., 2012). This was explained by the effect of reactive oxygen species (in this case hydrogen peroxide). In plasma generated by air as process gas, the formation of reactive oxygen species is higher than in plasmas generated by O_2_ and CO_2_, where instead a higher amount of ozone is released (Hertwig et al., 2017). Also hydrogen peroxide in particular was found to be formed in dielectric barrier discharges generated by air (Kalghatgi et al., 2012). Therefore, it is assumed, that the exposure of *P. verrucosum* to the reactive oxygen and nitrogen species generated by the plasma constituted a stressor, which resulted in a higher production of OTA. The plasma treatment for 3 min caused certainly more damage to the molds; therefore the amount of OTA was lower than after plasma treatment for only 1 min. Furthermore, the incubation time before the treatment was only 24 h, therefore the amount of OTA produced by *P. verrucosum* on the barley was lower and consequently less of the mycotoxin could be inactivated directly through the plasma. Additionally, the low storage temperature could increase the mycotoxin production by a simultaneous retardation of the mold growth (Sherwood and Peberdy, 1974).

In general, the variance of the produced OTA amounts was unexpectedly high, leading to the fact, that most results regarding the plasma effect on the OTA production of *P. verrucosum* were not significant. A reason therefore could be an inhomogeneous plasma treatment, although the distribution on the plasma plate appeared even. However, this assumption is contradicted by the low standard deviations of the total mold counts, leading to significant mold reductions. Another explanation could lie in the nature of the mold, provoking an unequal OTA production under stress conditions. However, the tendencies were clearly visible, leading to the implication that the process gas of the plasma treatment has a strong impact on the production of the mycotoxins. It can be assumed that these contradictory results would also appear with other plasma gasses and sources. This has to be considered when using plasma treatment for inactivation of molds, particularly for a large-scale industrial application. Dielectric barrier discharges principally have a promising design for scaling-up, allowing continuous processing approaches generally used in food industry. However, to realize the retention of the long-lived reactive species in a continuous process, which would lead to lower treatment times, will be one of the challenges in the future (Cullen et al., 2017).

## Conclusion

Three minutes treatment of barley inoculated with *A. niger* and *P. verrucosum* using plasma generated by dry air effectively reduced the total mold count by 2.5–3 log cycles after two weeks. However, since the hazardous aspect of these molds is their ability to produce mycotoxins, it is very important to consider the effect of plasma treatment also on the production of mycotoxins. Here, the various process gasses led to contradictory results. In the case of CO_2_ + O_2_ plasma, the OTA production of *P. verrucosum* was clearly reduced. When using CO_2_ as process gas, the OTA amount was nearly the same directly after the treatment but was increased, although not significantly, after two weeks of storage. Finally, the use of dry air for plasma generation resulted in a decreased OTA concentration when the incubation time before the treatment was five days and in an increased OTA amount when the barley was incubated only 24 h before the treatment. Therefore, the conditions including the incubation time and the process gas have to be taken into account when using plasma treatment to reduce the mold and mycotoxin concentration on grains and to avoid a stimulation of mycotoxin production. Additionally, it must always be carried out a combined examination of molds and their metabolite mycotoxin in order to correctly evaluate the success of the inactivation process.

## Author Contributions

JD conceived and designed the experiments, performed the experiments, analyzed and interpreted the data, and wrote the manuscript. OS conceived and designed the experiments, contributed reagents, material, analysis tools, or data, and proofread the manuscript. AR performed the experiments. PD analyzed and interpreted the data. AF conceived and designed the experiments, analyzed and interpreted the data, and proofread the manuscript.

## Conflict of Interest Statement

The authors declare that the research was conducted in the absence of any commercial or financial relationships that could be construed as a potential conflict of interest. The handling Editor declared a past co-authorship with the authors OS and AF.
